# Critical Incident Disclosing Behaviors and Associated Factors among Nurses Working in Amhara Region Referral Hospitals, Northwest Ethiopia: A Cross-Sectional Study

**DOI:** 10.1155/2021/8813368

**Published:** 2021-01-11

**Authors:** Yeshiambew Eshete, Bekele Tesfaye, Zewdu Dagnew, Demewoz Kefale, Demke Mesfin Belay, Binyam Minuye

**Affiliations:** ^1^Department of Nursing, College of Health Sciences, Debretabor University, Debretabor, Ethiopia; ^2^College of Health Sciences, Debremarkos University, Debremarkos, Ethiopia

## Abstract

**Background:**

Though the goal of healthcare institutions is patient safety, errors have been committed by healthcare providers. Incident reporting behavior enhances patient safety by reducing the repeated occurrence of errors in the health facility. Therefore, this study aims to identify incident disclosing behaviors and associated factors among nurses working in referral hospitals, Northwest Ethiopia.

**Methods:**

Institution-based cross-sectional study design was conducted among randomly selected 319 nurses working in referral hospitals of Amhara region from March 1–30, 2019. Data were collected using a self-administered structured questionnaire. Data were coded and entered into EpiData 4.2 software and exported to Statistical Package for Social Sciences version 25 for analysis. All variables with *p* value <0.25 during bivariable binary logistic regression analysis were considered for multivariable binary logistic regression analysis. Odds ratio along with 95% CI was estimated to measure the strength of the association. Level of statistical significance was declared at *p* value ≤0.05.

**Results:**

The proportion of nurses who reported incidents was 31.9% (95% confidence interval (CI), 27, 3)). Fear of administrative sanctions (adjusted odd ratio (AOR) = 0.45; 95% CI, 0.22, 0.90), fear of legal penalty (AOR = 0.27; 95% CI, 0.14, 0.50), lack of feedback (AOR = 0.29; 95% CI, 0.13, 0.66), nonsupportive environment (AOR = 0.27; 95% CI, 0.14, 0.52), and feel that reporting to colleague is easier (AOR = 2.65; 95% CI, 1.35, 5.20) were all found to be significant factors.

**Conclusions:**

The proportion of nurses who reported incidents was low. Fear of administrative sanctions, fear of legal penalty, lack of feedback, nonsupportive environment, and felling that reporting to colleagues was easier are found to be significant factors. Developing a system that encourages critical incident reporting behavior and provide protection from penalties for nurses to report incidents for the enhancement of patient safety and quality of care at each health facility and regional level is crucial.

## 1. Background

Incidence is an undesirable adverse event, injury, and a near miss that lead to patient's harm. Errors can occur in all stages in the process of care delivery [1–3]. Incident reporting is a tool that enables nurses to disclose incidents to others such as colleagues, patients, and concerned bodies (Administrative personnel's, supervisors, and directors). Incident reporting permits a retrospective window on the healthcare system by providing extremely valuable information for prevention of future errors, by identifying weaknesses of the system and detecting preventable events [4, 5]; however, many of the incidents are not reported.

Medical error is an inevitable and continuous problem in healthcare facilities. It increases from time to time and poses a great threat to the safety of patients and quality of care [2, 6]. According to 1999 Institute of Medicine (IOM) report, between 44,000 and 98,000 people die annually as a result of medical errors, and studies in the United States of America also estimated that the number of people died due to medical incident accounts 142,000 [7–9].

To minimize and avoid the repeated occurrence of incidents, healthcare facilities need to change policies and procedures that encourage nurses to report incidents. In addition, the protocol also creates an opportunity to enhance nurses to learn from mistakes, and this will also further enhance patient safety [10, 11]. Studies recommend that reoccurrence of errors can be prevented by the incident reporting system. However, lack of reporting of incidents by nurses as well as health facility leaders increases incidents in an alarming rate which affects patient safety throughout the world [12, 13].

Identification, documentation, and analyzing of actual or potential harm to patients as well as timely report is crucial for the development and functioning of safer healthcare systems which require combined changes of healthcare facility leaders and healthcare workers [14, 15]. Despite the significance of contribution of incident reporting to patient safety and quality of care, underreporting behaviors of nurses due to different factors such as sanction, deployment, and penalty are high. There are evidences regarding the burden of incident in developed countries, but little is known in developing countries such as Ethiopia. Therefore, the current study was intended to fill the information gap on incident reporting behavior of nurses working in Amhara region referral hospitals.

## 2. Methods

### 2.1. Study Design, Period, Setting, and Population

An institution-based cross-sectional study was conducted from March 1–30, 2019, at referral hospitals of Amhara region (Debre Markos Referral Hospital, Debre Birhan Referral Hospital, Dessie Referral Hospital, and Felege Hiwot Referral Hospital), Ethiopia. All nurses working in referral hospitals of Amhara regional state with ≥6 month experience were the target population. Those nurses who were giving free service and annual leave were excluded. Finally, 319 nurses were included in the study.

### 2.2. Data Collection Methods

Data were collected by using a structured self-administered English version questionnaire which was adapted from a study conducted by Engeda [10] with the authors' permission. The tool was composed of four parts, such as sociodemographic characteristics of nurses, levels of incident reporting, institutional factors, and perceived barriers of nurses to incident reporting. The data were collected by four nurses, two Bachelor of Science degree (BSc) nurse supervisors. Completeness of each recording format was checked before collecting the data.

### 2.3. Variables

#### 2.3.1. Dependent Variable

Incident disclosing behavior is the dependent variable.

#### 2.3.2. Independent Variables

- Sociodemographic characteristics: age, sex, nursing educational level, years of service as a nurse, working hours per week, and primary work area
- Institutional factors: training on incident reporting, availability of guideline/policy, and availability of reporting formats
- Perceived barriers: nonsupportive environment (culture of shame and blame), loss of prestige among colleagues, fear of legal or financial penalties, fear of administrative sanction, and lack of feedback

### 2.4. Operational Definitions

- Incident reporting behavior: nurses' action towards the error they encounter during the delivery of care to report or not report (disclose) to their colleague or concerned body [10, 16]
- High incident reporting behavior: nurses who choose the option “always” for each item of outcome measuring questions listed and score greater or equal to the mean [5]
- Low incident reporting behavior: nurses who choose one of the options “most of the time,” “sometimes,” “rarely,” or “not reported at all” for each item of outcome measuring questions listed above and score below to the mean [10]

### 2.5. Data Quality Control

The pretest was performed on 5% of the sample size one week before the actual data collection time. One day training was given for data collectors and supervisors on data collection tools and data collection procedures. Completeness of each data collection tool had been checked by the principal investigator and the supervisors in a daily base. Double data entry was performed by two data clerks, and the consistency of the entered data was cross-checked.

### 2.6. Data Processing and Analysis

The data were entered, coded, cleaned, and checked using EpiData statistical software version 4.2.0.0, and analysis was performed by using SPSS version 25 statistical software. Summation of the scores for the scale incident reporting behavior was made to determine the level of incident reporting. The data with the Likert scale were analyzed as categorical data (the scores for three outcome variables were summed up and then categorized). Descriptive statistics such as proportions, percentages, ratios, frequency distributions, and appropriate graphical presentations were used for describing the data. All variables with a *p* value ≤0.25 in the bivariable binary logistic regression analysis were entered into the final model for multivariable analysis. Variables with *p* values ≤0.05 were considered as statistically significant with the outcome variable.

### 2.7. Ethical Considerations

Ethical clearance was obtained from Debre Markos University, College of Health Sciences, and Institutional Health Research Ethics Review Committee (IHRERC). Then, official letters were written to each referral hospital for permission and support. Permission letter was obtained from the selected hospitals. Written consent was taken from each participant, and confidentiality was assured. The data collected was used for the study purpose. Privacy and anonymity was secured.

## 3. Results

### 3.1. Sociodemographic Characteristics of the Study Participants

In this study, a total of 288 nurses were participated in the study making a response rate of 90.3%, of which 56% (162) was male. The median age of nurses was about 28.5 with an interquartile range (IQR) of 26.5–32 years. Majority, 245 (85%) nurses were orthodox Christian. Almost all 276 (96%) were Amhara in ethnicity. The mean working hours per week was 54.07 (±9.84) (Table 1).

### 3.2. Incident Disclosing Behavior

A total of 288 nurses participated in the study, and the finding showed that overall incidents reporting behavior of nurses was 31.9% (95% CI, 27–37).

### 3.3. Factor Associated with Incident Reporting Behavior

A binary logistic regression was performed to identify the association between incident reporting behavior and independent variables. In bivariable logistic regression, age, sex, taking, training on incident reporting, availability of the reporting format, fear of administrative sanctions, fear of financial or legal penalty, lack of feedback, reporting to senior nurses, reporting to colleagues, reporting to medical director, working hours per week, develop a system to minimize repetition of error, religion, and nonsupporting (culture of shame or blame) environment met the criteria to be fitted into a multivariable analysis (*p* value ≤0.25).

Fear of administrative sanctions (AOR = 0.45, 95% CI, 0.22, 0.90), fear of legal penalties (AOR = 0.27, 95% CI, 0.14, 0.500), nonsupporting environment (culture of shame or blame) (AOR = 0.27, 95% CI, 0.14, 0.52), lack of feedback (AOR = 0.29, 95% CI, 0.13, 0.66), and reporting to colleague is easier (AOR = 2.65, 95% CI, 1.35, 5.20) were significantly associated with the outcome variable in multivariable analysis. Accordingly, nurses who feared administrative sanctions were 55% less likely to report incident as compared to those nurses who did not fear administrative sanctions. Beside, nurses who feared legal penalty and working in nonsupporting environment were 73% less likely to report incidents as compared to its counterpart. Moreover, nurses who believed that reporting to colleague is easier were 2.65 times more likely to report the incident than those nurses who did not believe reporting to colleague. Finally, providing feedback for nurse's increases incident reporting behavior of nurses by 61% (Table 2).

## 4. Discussion

Incident reporting systems in hospitals provide valuable data and information for improving patient safety through analysis of the nature and patterns of incidents that have been occurred; it can be used for learning and grounds for precautionary action in the future [14]. This study showed the incident reporting behavior and associated factors in referral hospitals.

The overall incident reporting behavior was 31.9% (95% CI, 27–37) which is higher than the study conducted in Gondar Comprehensive Specialized Hospital (25.4%) [10]. The difference between the study setting and period was contributed for the discrepancy. According to the Ethiopian hospital reform implementation guideline, an incident officer is assigned to each hospital to receive and investigate all incident reports, and a summary report of all incidents must be submitted to a quality assurance committee of each hospital [17].

But lower than the study conducted in Egypt (66.7%), Korea (53%), and Iran (50.26%) [3, 18, 19]. The reason for this discrepancy may be accounted for legal protection of reporters, blame for the reported errors in the working environment, and lack of personal confidence to withstand any consequence of error reporting. The result of a study from Iran showed that the reasons that nurses do not report incidents were fear, disagreement over the occurred mistake, administrative reactions to errors, and the effort needed for the reporting process [20].

Similarly, this study revealed that nurses who believed that there is lack of feedback after reporting were 71% less likely to report incidents than its counterpart which is in line with studies conducted in the USA and Turkey [21, 22]. This could be due to the fact that nurses believed in a lack of root cause analysis, which is used as a means to understand factors contributing to medical errors and identify systemic factors that contribute to errors. It is the action or response to the reporting that brings the change within an organization. Reporting of incidents should lead to an in-depth investigation to assess the etiological factors. Thus, incident reporting without a response from the concerned body leads to a sense of futility in the incident reporting period.

Nurses who feared legal penalties were 73% less likely to report incidents than those who did not fear legal penalties. This is consistent with the result of studies conducted in Egypt and Iran [3, 20]. This fear might be due to adverse consequences of reporting such as a malpractice lawsuit, losing patients' trust, emotional reactions of the patients and their relatives, or losing occupational position. An incident that harmed or did not harm the patient also negatively influences the reporting behavior of nurses. The result of the study showed that most of nurses reported that an incident report could be used against them and there would not be legal protection. Besides, the penal code of Ethiopia state that hurting a patient as a result of negligence or medical error is punitive even if it is unintentional [10].

This finding also showed that nurses who believed there is a nonsupportive environment (a culture of blame and shame) were 73% less likely to report incidents than those believed they worked in a supportive environment. This is in agreement with results of studies conducted in Saudi Arabia and Korea [23, 24] which stated that nonsupportive environment prevents nurses from reporting incidents. This could be attributed to the cultural practice of naming and shaming that involved in medical errors reporting during meetings by facility managers and head nurses. A nurse who reports errors is considered as incompetent by communities of the institutions. As a result of this environment, nurses favor not reporting than disclosing the mistake to the concerned body.

Nurses who feared administrative sanctions were 55% less likely to report incidents than those who did not fear administrative sanctions. This finding is in line with previous studies conducted in Ethiopia and Iran [10, 20]. This could be attributed to the fact that nurses feel insecure about their job and are afraid that they will have to face administrative sanctions such as economic and disciplinary measures that could be taken after committing and reporting an error and health facility administration's focus on the person who committed errors rather than a system in which medical errors can be registered and analyzed [19]. In the context of Ethiopia, job profile quality has a great value for future nurse's career but reporting of errors results in oral or written warning which is attached to their files, and this ends up demotion to dismissal from the organization. Due to those consequences, nurses try to hold the incident from reporting unless there is a system that protects them from such kind of administrative interference.

Nurses who believed that reporting to colleague is easy were 2.65 times more likely to report incidents than those who did not believe that reporting to a colleague is not easy. The possible reason could be colleagues are easily found in the nearby as well as need them to control the adverse effects of mistakes committed by nurses. On top of that, the consequences of medical errors are overwhelming and psychologically unhealthy for the practicing nurses as well. Therefore, colleague is the preferred one to obtain the solution for escaping from this tension and keeping the occurrence of incident confidentially.

### 4.1. Limitation of the Study

The study participants were only nurses and social desirability bias.

## 5. Conclusions

The proportion of nurses who report all type of incidents in all situations is low. But, improved reporting behavior was demonstrated when the incident is caught and corrected before affecting the patient. The study also found important factors that prevent them from reporting errors such as nonsupportive environment, fear of administrative sanction, fear of legal penalty, lack of feedback, and reporting to colleagues is easier.

## Figures and Tables

**Table 1:** Sociodemographic characteristics of nurses working in Amhara regional state referral hospitals, 2019 (*n* = 288).

Variable	Frequency	Percentage
Age		
20–29 years	168	58.3
30–39 years	87	30.2
40–49 years	25	8.7
50–60 years	8	2.8

Sex		
Male	164	56.9
Female	124	43.1

Marital status		
Single	103	35.8
Married	185	64.2

Religion of participant		
Orthodox Christian	245	85.1
Muslim	39	13.5
Protestant	2	1.4

Ethnicity of participant		
Amhara	276	95.8
Others	12	4.2

Educational level		
Diploma nurse	27	9.4
BSc nurse	253	87.8
MSc nurse	8	2.8

Position of nurse		
Head nurse	24	8.3
Staff nurse	264	91.7

Year of service as nurse		
1–5	146	50.7
6–10	91	31.6
11–15	23	8
16–20	18	6.3
21 and above	10	3.4

Working hours per week		
39–49 hours	73	25.4
50–60 hours	170	59
Above 60 hours	45	15.6

**Table 2:** Bivariable and multivariable analysis of incident reporting behaviors among nurses working in Amhara regional state referral hospitals, 2019 (*n* = 288).

Variable	Incident reporting		Odds ratio (95% CI)	
	Yes	No	COR	AOR
To whom reporting is easier to colleagues				
Yes	30	29	2.79 (1.55–5.02)	2.65 (1.35–5.20)^*∗*^
No	62	167	1	1
Reporting to senior nurse				
Yes	27	85	0.54 (0.22–0.92)	0.71 (0.48, 2.88)
No	65	111	1	
Reason to report to develop a system				
Yes	24	87	0.44 (0.26–0.76)	0.32 (0.12, 1.45)
No	68	109	1	
Organizational factors training on error reporting				
Trained	23	107	0.28 (0.16–0.48)	0.67 (0.23, 1.20)
Not trained	69	89	1	
Availability of report format				
Yes	25	38	1.55 (0.87–2.77)	1.34 (0.67, 2.8)
No	67	158	1	1
Perceived barrier to report nonsupportive environment				
Yes	20	97	0.28 (0.16–0.50)	0.27 (0.14–0.52)^*∗*^
No	72	99	1	1
Fear of administrative sanction				
Yes	16	86	0.27 (0.15–0.49)	0.45 (0.22–0.90)^*∗*^
No	76	110	1	1
Fear of legal penalty				
Yes	24	116	0.24 (0.14–0.42)	0.27 (0.14–0.50)^*∗*^
No	68	80	1	1
Lack of feedback				
Yes	12	47	0.48 (0.24–0.95)	0.29 (0.13–0.66)^*∗*^
No	80	149	1	1

^*∗*^ *p* value <0.05.

## Data Availability

All relevant data are included within the article and are available from the corresponding author upon request.

## Abbreviations

AOR: Adjusted odds ratio
DBRH: Debre Birhan Referral Hospital
DMRH: Debre Markos Referral Hospital
DRH: Dessie Referral Hospital
FHCSH: Felege Hiwot comprehensive specialized hospital
IRS: Incident reporting system
IOM: Institute of Medicine
ME: Medical error
SD: Standard error
WHO: World Health Organization.
