# Corrigendum to “Critical Incident Disclosing Behaviors and Associated Factors among Nurses Working in Amhara Region Referral Hospitals, Northwest Ethiopia: A Cross-Sectional Study”

**DOI:** 10.1155/2021/9842972

**Published:** 2021-04-27

**Authors:** Yeshiambew Eshete, Bekele Tesfaye, Zewdu Dagnew, Demewoz Kefale, Demeke Mesfin Belay, Binyam Minuye

**Affiliations:** ^1^Department of Nursing, College of Health Sciences, Debretabor University, Debretabor, Ethiopia; ^2^College of Health Sciences, Debremarkos University, Debremarkos, Ethiopia

In the article titled “Critical Incident Disclosing Behaviors and Associated Factors among Nurses Working in Amhara Region Referral Hospitals, Northwest Ethiopia: A Cross-Sectional Study” [1], there was a spelling error in author Demeke Mesfin Belay's name in the author list, where “Demke Mesfin Belay” should have read “Demeke Mesfin Belay.” This is corrected as shown in the author details above.
